# A Clinical and Epidemiological Investigation of the First Reported Human Infection With the Zoonotic Parasite *Trypanosoma evansi* in Southeast Asia

**DOI:** 10.1093/cid/ciw052

**Published:** 2016-02-07

**Authors:** Nguyen Van Vinh Chau, Le Buu Chau, Marc Desquesnes, Stephane Herder, Nguyen Phu Huong Lan, James I. Campbell, Nguyen Van Cuong, Benjarat Yimming, Piangjai Chalermwong, Sathaporn Jittapalapong, Jose Ramon Franco, Ngo Tri Tue, Maia A. Rabaa, Juan Carrique-Mas, Tam Pham Thi Thanh, Nga Tran Vu Thieu, Alessandra Berto, Ngo Thi Hoa, Nguyen Van Minh Hoang, Nguyen Canh Tu, Nguyen Khac Chuyen, Bridget Wills, Tran Tinh Hien, Guy E. Thwaites, Sophie Yacoub, Stephen Baker

**Affiliations:** 1 Hospital for Tropical Diseases, Ho Chi Minh City, Vietnam; 2 Centre de Coopération Internationale en Recherche Agronomique pour le Développement (CIRAD), UMR Intertryp, Montpellier, France; 3 Department of Parasitology, Faculty of Veterinary Medicine, Kasetsart University, Bangkok, Thailand; 4 UMR 177 Intertryp IRD/CIRAD, Montpellier, France; 5 Oxford University Clinical Research Unit, Wellcome Trust Major Overseas Programme, Ho Chi Minh City, Vietnam; 6 Centre for Tropical Medicine and Global Health, Nuffield Department of Clinical Medicine, Oxford University, United Kingdom; 7 Department of Control of Neglected Tropical Diseases, World Health Organization, Geneva, Switzerland; 8 Regional Animal Health Laboratory 5; 9 Subdepartment of Animal Health, Buon Ma Thuot, Dak Lak, Vietnam; 10 Department of Medicine, Imperial College London, Hammersmith Campus; 11 Department of Pathogen and Molecular Biology, London School of Hygiene and Tropical Medicine, United Kingdom

**Keywords:** Vietnam, zoonosis, *Trypanosoma evansi*, case investigation, emerging infections

## Abstract

***Background.*** *Trypanosoma* is a genus of unicellular parasitic flagellate protozoa. *Trypanosoma brucei* species and *Trypanosoma cruzi* are the major agents of human trypanosomiasis; other *Trypanosoma* species can cause human disease, but are rare. In March 2015, a 38-year-old woman presented to a healthcare facility in southern Vietnam with fever, headache, and arthralgia. Microscopic examination of blood revealed infection with *Trypanosoma*.

***Methods.*** Microscopic observation, polymerase chain reaction (PCR) amplification of blood samples, and serological testing were performed to identify the infecting species. The patient's blood was screened for the trypanocidal protein apolipoprotein L1 (APOL1), and a field investigation was performed to identify the zoonotic source.

***Results.*** PCR amplification and serological testing identified the infecting species as *Trypanosoma evansi.* Despite relapsing 6 weeks after completing amphotericin B therapy, the patient made a complete recovery after 5 weeks of suramin. The patient was found to have 2 wild-type *APOL1* alleles and a normal serum APOL1 concentration. After responsive animal sampling in the presumed location of exposure, cattle and/or buffalo were determined to be the most likely source of the infection, with 14 of 30 (47%) animal blood samples testing PCR positive for *T. evansi*.

***Conclusions.*** We report the first laboratory-confirmed case of *T. evansi* in a previously healthy individual without APOL1 deficiency, potentially contracted via a wound while butchering raw beef, and successfully treated with suramin. A linked epidemiological investigation revealed widespread and previously unidentified burden of *T. evansi* in local cattle, highlighting the need for surveillance of this infection in animals and the possibility of further human cases.

## Abstract (precis)

We show that the bovid-associated parasite *Trypanosoma evansi* is endemic in Vietnam and has zoonotic potential. Our study describes the first laboratory-confirmed human case of *T. evansi* in a previously healthy individual without apolipoprotein L1 deficiency.

Southeast Asia is a hotspot for emerging and remerging infectious diseases [1]. Over the last decade, Vietnam in particular has been an epicenter for such infections [2]. The country has a human population of >90 million, some of the highest densities of farmed livestock in Asia, and a substantial burden of infectious diseases, many of which occur without an etiological diagnosis [3]. More than half of Vietnam's population resides in rural areas, and most participate in small-scale animal production, which likely facilitates the transfer of pathogens from animals into humans (zoonotic transmission). Therefore, we predict that zoonotic infections occur frequently in rural locations but do not trigger onward human-to-human transmission and often fail to be diagnosed [4]. Furthermore, because of limited diagnostic capacity in provincial animal health facilities, conclusive epidemiological investigations of zoonotic infections in potential animal sources are expensive, challenging, and rarely performed.

*Trypanosoma* is a genus of unicellular parasitic flagellate protozoa within the class Kinetoplastida. The genus has several members, the majority of which require transmission between different hosts to complete their life cycle. *Trypanosoma* infections are responsible for a large burden of infectious disease in humans globally [5]. The major zoonotic parasites, *Trypanosoma brucei* species (*T. brucei* subspecies *gambiense* and *T. brucei* subspecies *rhodesiense*) and *Trypanosoma cruzi* cause a substantial burden of sleeping sickness and Chagas disease in sub-Saharan Africa and Latin America, respectively. Other *Trypanosoma* species, such as *T. lewisi*, *T. brucei* subspecies *brucei*, *T. congolense*, and *T. evansi*, can also cause disease in humans, but are rare [6]. The majority of these atypical human cases of *Trypanosoma* infections are due to *T. lewisi* and *T. evansi. Trypanosoma lewisi* is a ubiquitous, nonpathogenic parasite of rats, and is considered to be a human pathogen, although there have been only a limited number of reported cases [7]. *Trypanosoma evansi* is associated with acute disease in camels and horses (surra) and chronic disease in cattle and buffalo, and can be found in South America, North Africa, the Middle East, and South and Southeast Asia [8].

The only human case of *T. evansi* with molecular confirmation of the infecting species occurred in India in 2005 [9]. This patient had a deficiency of apolipoprotein L1 (APOL1) [10], a component of human serum with trypanocidal activity [11]. Four other probable cases have been reported worldwide [8], 3 in India and 1 in Egypt, but molecular parasite speciation was not performed. Here we describe a clinical, parasitological, and epidemiological investigation of the first confirmed human infection of *T. evansi* in a previously healthy individual without APOL1 deficiency in Asia.

## CASE REPORT

On 19 March 2015, a 38-year-old Vietnamese woman presented to a healthcare facility in Dong Nai province in southern Vietnam (approximately 30 km east of Ho Chi Minh City) with 18 days of fever, headache, and arthralgia. She did not seek medical advice during this period but self-treated with antipyretics. She worked in a footwear factory, had given birth on 23 November 2014, and was breastfeeding a healthy infant; her past medical history was unremarkable with no known underlying medical condition. Her only account of recent travel was to Krong Pac district in Dak Lak, a rural province in Vietnam's Central Highlands, from 8 February to 2 March, to visit relatives over the lunar New Year holiday. She had never traveled outside Vietnam.

On presentation at the provincial hospital, she was treated presumptively for malaria with dihydroartemisinin-piperaquine. The following day she remained febrile and was transferred to the Hospital for Tropical Diseases (HTD) in Ho Chi Minh City. On admission at HTD, she had a severe headache but no additional neurological symptoms or signs, and her Glasgow Coma Scale score was 15/15. Her temperature was 39.5°C, respiratory rate was 22 breaths/minute, pulse was 100 beats/minute, and blood pressure was 110/70 mm Hg. Cardiorespiratory examination was normal and abdominal examination revealed nontender hepatomegaly.

**Figure 1.**
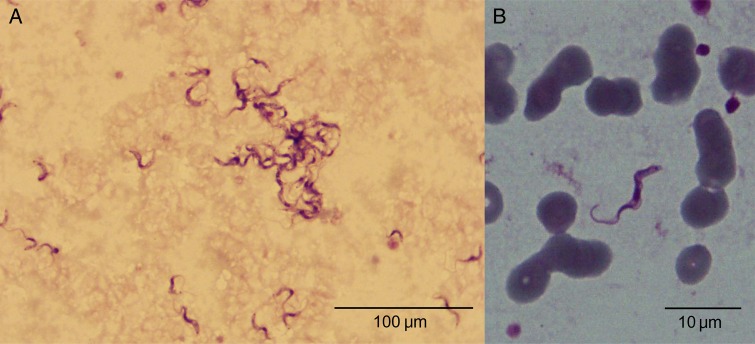
Giemsa-stained blood sample showing *Trypanosoma evansi* in the blood of a 38-year-old Vietnamese woman at ×100 (*A*) and ×1000 (*B*) magnification, with typical forms of the subgenus *Trypanozoon* parasites (total length 20–30 µm, free flagellum, central nucleus, large undulating membrane, subterminal small kinetoplastids [<0.8 µm], and a sharp posterior extremity).

Initial blood tests showed pancytopenia and mild renal impairment with raised liver aminotransferases and hypoalbuminemia (Table 1); electrolytes, lactate dehydrogenase, creatine phosphokinase, and troponin I were within normal ranges. Microbiological cultures performed on blood, urine, and cerebrospinal fluid (CSF) did not produce any significant growth; CSF cell count, protein, and glucose concentrations were within normal ranges. Serological tests for hepatitis B, hepatitis C, and human immunodeficiency virus were negative. A blood film, stained with Giemsa and examined by microscopy, was negative for malaria; however, numerous unicellular flagellate protozoa, with the distinctive morphology of *Trypanosoma* and with an estimated concentration of >50 000 parasites/µL, were identified (Figure 1). Motile parasites were also observed by direct microscopic examination of the blood (Supplementary Data). Chest radiography and electrocardiography were normal, and abdominal ultrasound confirmed hepatomegaly (164 mm) and splenomegaly (117 mm).

**Table 1.** Timeline of the Clinical Laboratory Investigations and Results of a Human *Trypanosoma evansi* Infection—Vietnam, 2015

Test	Normal Range	First Admission	First Discharge	Second Admission	Second Discharge	Follow-up
Date	…	19 March	28 March	15 May	12 June	13 July
WBC count, cells/µL	4500–11 000	**1680**	5250	**3260**	**2880**	**4170**
Neutrophil count, cells/µL	2000–7500	**530**	2226	**1330**	**1630**	2247
Hemoglobin, g/dL	12–16	7.7	12.2	7.2	**10.7**	12.5
Platelet count, ×10^9^ cells/L	150–400	**97**	270	**51**	183	196
Procalcitonin, ng/mL	<0.15	**32.25**	ND	**43.7**	ND	ND
Creatinine, mmol/L	60–115	**143**	**48**	**139**	66	66
Ferritin, ng/mL	15–200	**1453**	**368**	**291**	ND	ND
Albumin, g/L	35–55	**26.1**	42.8	**24.7**	38	ND
Bilirubin, µmol/L	5.1–20.5	7.9	**2.9**	**61.4**	8.2	ND
AST, U/L	0–35	**78**	27	**89**	20	18
ALT, U/L	0–35	**449**	**40**	**186**	22	19

Values outside the normal range are shown in bold.

Abbreviations: ALT, alanine aminotransferase; AST, aspartate aminotransferase; ND, not determined; WBC, white blood cell.

First-line antitrypanosomal drugs (eg, pentamidine and suramin) are not available in Vietnam. Consequently, the patient was treated with amphotericin B (0.7 mg/kg/day) for 7 days, due to its reported trypanocidal activity [12]. She was afebrile after 12 hours of treatment. After 24 and 48 hours of treatment, blood films demonstrated a fall in trypanosoma parasitemia from 11 880 parasites/µL to 5900 parasites/µL, respectively. No parasites were observed in blood films by the fourth day of treatment. White blood cell and platelet counts improved, while hemoglobin dropped to 6.6 g/dL. She was discharged without symptoms and with normal blood values on the 10th day after admission (Table 1). The patient was recalled for follow-up 4 weeks after discharge; she remained asymptomatic with normal blood values, and no parasites were visible upon microscopy.

Six weeks after hospital discharge (15 May), the patient returned to HTD with 2 days of fever, headaches, nausea, and vomiting. Blood tests again demonstrated pancytopenia, raised liver enzymes, and hypoalbuminemia (Table 1). An ultrasound showed hepatomegaly of 167 mm and splenomegaly of 126 mm, and a peripheral blood smear identified >70 000 parasites/µL. CSF examination was normal. By this time, suramin had been sourced from the World Health Organization (WHO), and she was treated with 1 g weekly for 5 weeks. The fever resolved within 8 hours of the first dose, and the blood smear was negative within 12 hours. The patient was discharged and completed suramin treatment without complications. She was followed up monthly for 6 months with repeated clinical examination and blood films. Further investigations to identify the infecting parasite, the patient's serological responses and susceptibility, and the potential source were subsequently undertaken.

## METHODS

### Parasite Identification and Genotyping

Subgenus identification of the trypanosomes was performed by morphology and morphometry under microscopic examination of the parasite using Giemsa-stained blood smears. To detect parasite DNA in blood samples, the following primers were used for polymerase chain reaction (PCR) amplification: Tryp1 (generic for trypanosomes) [13], NRP and TBR (*Trypanozoon* subgenus) [14], EVA [15], TEPAN [16], TE2249/50 [17], RoTat1.2 F/R [18] (*T. evansi*/*T. equiperdum* specific), EVAB1/2 [19, 20] (*T. evansi* type B specific), and Lew [21] (*T. lewisi* species specific). DNA was extracted using the QIAamp DNA Blood Mini Kit (Qiagen) following the manufacturer's recommendations. PCR amplifications were performed in volumes of 50 μL using amplification conditions described in the cited publications.

For parasite characterization, amplification and sequencing of a 234-bp polymorphic fragment of the small subunit (SSU) 18S ribosomal DNA (rDNA) was performed as previously described [22]. Novel nucleotide sequences of the partial SSU ribosomal RNA gene of Vietnamese *T. evansi* were deposited in GenBank under the following accession numbers: KT844943, *T. evansi* isolate T1 human Vietnam first infection; KT844944, *T. evansi* isolate T2 cattle Vietnam; KT844945, *T. evansi* isolate T3 cattle Vietnam; and KT844946, *T. evansi* isolate T4 human relapse Vietnam.

### Serological Testing

The card agglutination test *T. evansi* (CATT/*T. evansi*) (Institute of Tropical Medicine, Antwerp, Belgium) is for the serodiagnosis of animal trypanosomiasis caused by *T. evansi* [23], and can detect anti–*T. evansi* antibody in blood, serum, and plasma [24]. Assays were performed following the manufacturer's recommendations (serum dilution 1:4), and positive serum samples were further tested in serial dilutions to provide a semiquantitative estimate of anti–*T. evansi* antibody in the patient's serum. A previously described *T. evansi* enzyme-linked immunosorbent assay (ELISA) was also performed using the first patient sample as positive control [25].

### APOL1 Testing

The patient underwent *APOL1* genotyping using targeted PCR amplification and sequencing as previously described [10]. Total genomic DNA was extracted from blood samples using the Nucleon Genomic DNA Extraction Kit (GE Healthcare). PCR was used to amplify the *APOL1* gene using the primers F-5′ AGCCACCACACCGAGCCAAAACTGC and R-5′ AGCACAAGAAAGAAGCTTACAGGGG to amplify a 783-bp region of *APOL1* on chromosome 22q13.1. PCR amplicons were cloned into pCR2.1^R^ Topo and sequenced in both directions using BigDye sequencing on an ABI3700XL DNA sequencer (Applied Biosystems). Quantitative Sandwich ELISAs (USCN Life Sciences) were performed in duplicate according to the manufacturer's instructions to measure the concentration of APOL1 in patient serum and the serum of 12 healthy anonymous Vietnamese volunteers.

### Field Investigation

We conducted a census of cattle and buffalo farms located within a 0.5-km radius of the patient's relatives' household. From each sampled animal, blood was collected by ear puncture and from the jugular vein into ethylenediaminetetraacetic acid (EDTA) tubes. EDTA blood was used to prepare both thick and thin blood smears that were fixed and stained using Giemsa. Buffy coat from centrifuged microcapillary tubes and Giemsa-stained smears were examined at ×100 magnification for the presence of trypanosomes. EDTA blood from the jugular vein was centrifuged and the buffy coat and plasma were separated; the former was used for molecular testing, and the latter for serological testing. Rats were trapped on several of the sampled farms, and blood samples were tested by serology and PCR amplification. Staff from the sub–Department of Animal Health and the Regional Animal Health Laboratory performed the animal sampling. Serological tests were performed using CATT on plasma from all species; ELISAs were performed on bovids only.

### Ethical Approval

Written informed consent was obtained from the patient involved in this investigation. Permission for the field investigation was covered by existing protocols for an ongoing study of zoonotic infections in Vietnam (VIZIONS) [4], which were approved by HTD and the Oxford Tropical Research Ethics Committee.

## RESULTS

### Patient Serological Response, *Trypanosoma* Identification, and APOL1 Testing

Six plasma samples from the patient (primary presentation, relapse, and late follow-up) gave positive agglutinations using the CATT/*T. evansi* test and were positive at >1:512, 1:256, and 1:16 dilutions on primary presentation, convalescence, and relapse, respectively (Table 2). Posttreatment follow-up samples provided evidence for decreasing antibody concentrations, testing positive at 1:8, 1:8, and 1:4 dilutions at 1, 2, and 4 months after treatment, respectively. Whole blood from the patient was additionally subjected to PCR amplification to confirm the infecting species. The resulting amplifications tested positive for *Trypanozoon* and for *T. evansi*, and negative for *T. lewisi* and *T. evansi* type B (Table 2). It was concluded the patient was infected with *T. evansi* type A [13].

**Table 2.** Laboratory Confirmation of *Trypanosoma evansi* Infection—Vietnam, 2015

Date	Sample	Microscopy	CATT	CATT Titration	PCR Amplification^a^						
					Tryp1	EVA	NRP	TE2249/50	Rotat	TEPAN	Lew
19 March	First admission	+	+++	1:512	+	+	+	+	+	+	−
21 April	Follow-up	−	+++	1:256	−	−	−	−	−	−	−
15 May	Second admission	+	+	1:16	+	+	+	+	+	+	−
08 June	Follow-up	−	+	1:8	−	−	−	−	−	−	−
20 July	Follow-up	−	+	1:8	−	−	−	−	−	−	−
14 Sept	Follow-up	−	+	1:4	−	−	−	−	−	−	−

Abbreviations: CATT, card agglutination test *T. evansi*; PCR, polymerase chain reaction.

^a^ Tryp1 (generic for trypanosomes) [13], NRP and TBR (*Trypanozoon* subgenus) [14], EVA [15], TEPAN [16], TE2249/50 [17], RoTat1.2 F/R [18] (*T. evansi/T. equiperdum* specific), EVAB1/2 [19, 20] (*T. evansi* type B specific), and Lew [21] (*T. lewisi species* specific).

The APOL1 concentration in the patient's serum was 3394 ng/mL; the APOL1 concentrations in 12 healthy Vietnamese volunteers ranged from 1056 to 7400 ng/mL (median, 3094 ng/mL [interquartile range, 2754–4611 ng/mL]). Furthermore, the patient was confirmed to have 2 wild-type *APOL1* alleles (ie, no mutations in codon 142 or 266) upon amplification and sequencing of the gene encoding APOL1 [10].

### Field Investigation

Potential sources of *Trypanosoma* infection include horses, dogs, cattle, and buffaloes. The patient did not report any direct contact with animals while in Dak Lak province, but recollected buffalo and cattle in the area. She did not recall any bites from animals or insects, but did recollect inflicting a knife wound to her finger on 24 February while butchering locally reared raw beef.

**Figure 2.**
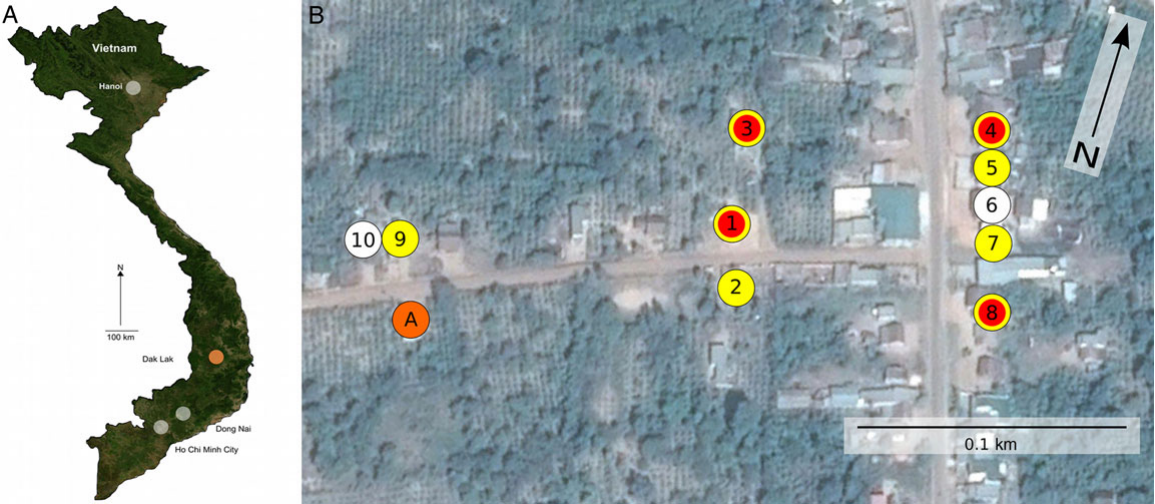
Map of sampled field location in Dak Lak province in central Vietnam. *A*, Map of Vietnam with the locations of Ho Chi Minh City, Dong Nai, and Dak Lak provinces highlighted. *B*, Map of farms sampled in response to the human case of *Trypanosoma evansi.* The orange circle (A) shows the location of the patient's house. The 10 sampled farm locations are shown and coded as follows: white, no evidence of trypanosomes; yellow, polymerase chain reaction and/or serological evidence of *T. evansi* in cattle or buffalo; red, microscopic evidence of *Trypanosoma* species in cattle or buffalo blood.

A total of 10 cattle and buffalo farms were identified within a 0.5-km radius from the patient's relatives' household in Krong Pac. These farms contained a total of 121 animals (37 chickens, 35 cattle, 27 buffaloes, 17 pigs, 4 dogs, and 1 cat) and 65 people (Figure 2). Blood was taken from 22 randomly selected cattle and 8 buffaloes. On microscopic examination, 3 of 22 (14%) blood samples from cattle on 3 different farms were found to contain *Trypanosoma* species; parasites were not observed in the blood of other animals.

PCR amplification for trypanosomes was performed on template DNA extracted from the blood of the sampled animals. Nine of 22 (41%) and 5 of 8 (63%) blood samples from cattle and buffalo, respectively, produced PCR amplicons indicative of *T. evansi* infection*.* Furthermore, 17 of 22 (77%) cattle blood samples and 6 of 9 (67%) buffalo blood samples had appreciable antibody against *T. evansi* by CATT and/or ELISA. Notably, on further questioning, farmers on 4 of 10 sampled farms (including all 3 of the farms with cattle that had a microscopy-confirmed parasitemia) reported recent unexplained illness in their cattle characterized by wasting, paralyzed limbs, and death, again indicative of *T. evansi* infection.

A comparison between four 18S rDNA sequences that were generated (2 from the patient's blood and 2 from cattle) exhibited 99.5% DNA homology over 234 bp. The 2 animal samples were additionally PCR positive for Rotat1.2 and negative for EVAB, thus suggesting *T. evansi* type A infection in both the patient and the sampled cattle.

## DISCUSSION

We present the first reported case of human *T. evansi* infection in Southeast Asia. *Trypanosoma evansi* is a zoonotic parasite that can be commonly found in bovine animals in tropical regions, although human disease is exceptionally rare [6]. The patient described here had no previously described immunological risk factors for *Trypanosoma* infection. Two mutations within the gene encoding apolipoprotein L1 (*APOL1*), in codons 142 and 266, have been previously associated with *T. evansi* susceptibility in an Indian patient [10]. APOL1 is a ubiquitous component of high-density lipoprotein found in plasma that kills trypanosomes by lysosome swelling [26]. Our patient did not have the genetic mutations associated with APOL1 deficiency, and serum concentrations were comparable to 12 healthy Vietnamese controls. Animal models suggest there may be other susceptibility factors to *Trypanosoma* infections, including increased host arginase activity and nitric oxide bioavailability [27], defective type I interferon responses, and deficiencies in the complement lectin pathway factors [28, 29]. The only identified risk factor was that the patient was 2–3 months postpartum at the time of exposure, which is a well-known risk factor for many infectious diseases.

There are no previous reports describing *T. evansi* treatment with amphotericin B and, although administration here correlated with defervescence and parasite clearance, it is difficult to conclude whether the drug or a natural immunological response induced this. Data from a murine *T. cruzi* model suggested that amphotericin B may have a trypanostatic action, but poor trypanocidal activity [12]. As our patient relapsed 6 weeks later, the efficacy of amphotericin B for *T. evansi* therapy remains ambiguous. Treatment with suramin, however, resulted in rapid and prolonged cure. Suramin is currently the first-line recommended treatment for early-stage *T. b. rhodesiense* and had been used successfully in treating human *T. evansi* previously [9]. Our case provides additional evidence for suramin's effectiveness against this parasite. Furthermore, a decrease in antibody response was also observed with CATT titers falling from 1:16 at the time of the treatment to 1:4 four months after infection. However, this CATT titer did not increase on relapse, suggesting a lack of immunoglobulin M on secondary parasitemia.

The rapid epidemiological investigation accompanying this first human *T. evansi* infection in Southeast Asia is testament to Vietnam's coordinated public and animal healthcare systems and links between clinicians and researchers. The rapid testing of >50 animals of different species in 10 different farms within a 0.5-km radius of the patient's residence found that a substantial proportion of bovids in the farms surrounding the patient's home had PCR-confirmed *T. evansi* infection (77% of bovids additionally had serological evidence of the infection). Furthermore, the parasites detected in the patient (primary infection and relapse) and parasites identified in bovids were *T. evansi* type A. Additional sequencing of the polymorphic fragment of the SSU 18s rDNA demonstrated a high degree of homology between the animal and human parasites, implicating local bovids as the most likely source of the infection.

*Trypanosoma evansi* is known to be endemic in Vietnam, and seroprevalences approaching 30% have been described in bovids and horses in the Red River Delta; outbreaks in central and southern Vietnam have been acknowledged within Vietnam [30]. However, the magnitude of the problem in terms of geographic distribution and prevalence in cattle/buffalo in Vietnam is currently undetermined and likely underestimated. The unexplained death of several bovines in the patient's locality was likely due to surra as indicated by the clinical signs and the prevalence of *T. evansi* in these species; these findings also imply that the parasite has been circulating undetected for some time. The endemicity of *T. evansi* may have economic consequences, but it also highlights the potential for further human cases. The precise route of transmission in this patient remains uncertain; however, direct inoculation through a hand wound, as was described for the previous case in India [9], is the most reasonable explanation.

In conclusion, we have described the first human case of *T. evansi* infection reported in Asia in a previously healthy individual without APOL1 deficiency. Subsequent field investigations demonstrated a high prevalence of bovids in the immediate environs of the patient with clinical and molecular evidence of *T. evansi* infection. The infection was most probably acquired through direct wound inoculation from meat of infected cattle. Further research is required to better understand this zoonotic pathogen, including host susceptibility factors, potential vectors, and therapeutic options for both human and animal infections.

## Supplementary Data

Supplementary materials are available at http://cid.oxfordjournals.org. Consisting of data provided by the author to benefit the reader, the posted materials are not copyedited and are the sole responsibility of the author, so questions or comments should be addressed to the author.

Supplementary Data
