# Supplementary material for: A Clinical and Epidemiological Investigation of the First Reported Human Infection With the Zoonotic Parasite Trypanosoma evansi in Southeast Asia
Source: Clin Infect Dis. 2016 Feb 7;62(8):1002–8. doi: 10.1093/cid/ciw052 (PMC4803109; doi:10.1093/cid/ciw052)
Supplement: Supplementary Data [file supp_62_8_1002__index.html]

A clinical and epidemiological investigation of the first reported human infection with the zoonotic parasite Trypanosoma evansi in Southeast Asia — A Clinical and Epidemiological Investigation of the First Reported Human Infection With the Zoonotic Parasite Trypanosoma evansi in Southeast Asia — A Clinical and Epidemiological Investigation of the First Reported Human Infection With the Zoonotic Parasite Trypanosoma evansi in Southeast Asia — Supplementary Data 

# A Clinical and Epidemiological Investigation of the First Reported Human Infection With the Zoonotic Parasite *Trypanosoma evansi* in Southeast Asia

## Supplementary Data

Supplementary Data

- Supplementary Data - Docx file
- Supplementary Video - mov file
