# Supplementary material for: A Clinical and Epidemiological Investigation of the First Reported Human Infection With the Zoonotic Parasite Trypanosoma evansi in Southeast Asia
Source: Clin Infect Dis. 2016 Feb 7;62(8):1002–8. doi: 10.1093/cid/ciw052 (PMC4803109; doi:10.1093/cid/ciw052)
Supplement: Supplementary Data [file supp_ciw052_ciw052supp.docx]

**Supplementary data.** Film of *Trypanosoma evansi* in the blood of patient
